# Health status of female Moldovan migrants to Italy by health literacy level and age group: a descriptive study

**DOI:** 10.1186/s12889-020-09582-9

**Published:** 2020-10-02

**Authors:** Francesca Alice Vianello, Federica Zaccagnini, Carlo Pinato, Pietro Maculan, Alessandra Buja

**Affiliations:** 1 Department of Philosophy, Sociology, Pedagogy and Applied Psychology, University of Padua, Piazza Capitaniato 3, 35129 Padova, Italy; 2 Melanoma and Sarcoma Unit, Veneto Institute of Oncology, IOV-IRCCS, via Gattamelata 64, 35128 Padova, Italy; 3 Department of Cardiological, Thoracic and Vascular Sciences, and Public Health, University of Padua, Via Loredan 18, 35128 Padova, Italy; 4 Laboratory for Assessing Health Care Services and Health Promotion, Hygiene and Public Health Unit, Dept. of Cardiological, Thoracic and Vascular Sciences, and Public Health, University of Padova, Via Loredan, 18, 35131 Padova, Italy

**Keywords:** Health literacy, Migrant workers, Moldovan women, Migrant women, Primary health care services, Health status

## Abstract

**Background:**

Migration flows from Eastern Europe to Italy have been large and continue to grow. The purpose of this study was to examine the health status of a population of Moldovan migrant women, and their access to health care services in northern Italy, by age group and health literacy level.

**Methods:**

We administered an ad-hoc questionnaire to adult Moldovan women. A bivariate analysis was conducted to test the association between health literacy and age groups with other variables (lifestyles, symptoms and diseases, access to health services). A stepwise logistic regression analysis was run to test the association between access to primary care and health literacy. Moreover, the study compare Moldovan women data with a sample of Italian women of the same age range living in North-Eastern region.

**Results:**

Our sample included 170 Moldovan women (aged 46.5 ± 12.3) in five occupational categories: home care workers (28.2%); cleaners (27.1%); health care workers (5.9%); other occupations (28.8%); and unemployed (10%). Active smokers were twice as prevalent among the women with a low health literacy. Health literacy level also determined access to primary healthcare services. For all age groups, the Moldovan sample reported a higher prevalence of allergies, lumbar disorders and depression than the Italian controls.

**Conclusions:**

The reported prevalence of some diseases was higher among Moldovan migrant women than among Italian resident women. Health literacy was associated with the migrant women’s lifestyle and the use of primary health care services, as previously seen for the autochthonous population.

## Background

International migration is one of the most predominant issues of our times. The migration of workers from Eastern to Southern Europe began in the late 1980s, after the dissolution of the former communist regimes, and increased enormously in subsequent decades [1, 2]. In Europe as a whole, many migrants from Eastern Europe have satisfied the increasing demand for a cheap labor force in low-skill and high-strain jobs. To give an example, only 6% of the domestic workers registered with the Italian National Social Security Institute in the early 1980s were of migrant origin, whilst the figure had reached 72% by 2006 [3, 4]. Moldovan migration, in particular, is highly feminized, and the flows from Ukraine and the Republic of Moldova largely consist of women who are independent migrants [5–7].

Worldwide, it has been reported that migrant workers in certain living and economic conditions may not seek health care - supposedly due to a limited knowledge of their rights or to cultural or language barriers [8]. This seems to be particularly true in the case of work-related diseases or injuries [9], and even applies to migrant women with a good formal education [5, 6]. It has also been established internationally that, for people to use health care services appropriately, they need to be able to access and understand health-related information, as captured in the concept of “health literacy”. This type of competence depends directly on a given individual’s language, education and culture. It is often described as an individual’s capacity both to process health-related information and actively choose a healthy behavior, and to actively and appropriately interact with health care services [10, 11].

Moldova’s worker migration flow is reportedly one of the highest in the world, with approximately 25% of the country’s economically active population in 2010 returning from working abroad, still working abroad, or intending to work abroad, according to the “Moldovan Labor Force Survey” [12]. Studies on the health status of these people and in particular of female migrants, and on their use of health care services in their destination countries, are lacking, however. Recognizing this target population’s health problems and ascertaining their inclination to use public health services could be useful in order to establish an effective program of health promotion and disease prevention for this particular migrant group.

Hence this descriptive study to examine the health status of a sample of Moldovan migrant women, and their recourse to health care services in Padova, a province in north-eastern Italy, by age group and health literacy level.

## Methods

### Context

The Italian National Health System (NHS) is a mainly public system financed by general taxation. All residents registered with the country’s NHS (be they Italians or regular immigrants) can access all healthcare services free of charge or by paying a small fee, and they are assigned a general practitioner (GP) of their choice. Italy is one of five of the 27 EU Member States to freely provide immigrants with much the same range of services as Italian nationals [13].

### Moldovan migration to Italy

The Republic of Moldova is not a member of the European Union, so its citizens cannot move freely within the EU. They need an appropriate document to cross its borders. Nonetheless, Moldovan migration to Italy has been quite considerable, and has increased in recent times. As at January 1st, 2019, there were 128,979 Moldovan citizens legally residing in Italy, and 66% of them (85,431) were women. In the province of Padua (in north-eastern Italy), their prevalence is akin to the national one, with 63% of women out of a total of 9866 Moldovan migrants. These Moldovans now account for 1% of the resident population (937,908) of the province of Padua [13].

### Sample

Our study population consisted of adult Moldovan women who understood Italian, recruited from February to June 2019 by a convenience sampling, since it was impossible to carry out a random sampling on Moldovan women residing in Padua because of privacy law. Questionnaires were administered face to face in the Italian language at various times and at four different venues in Padua, following the time-space method for sampling hard-to-reach populations [14, 15], though we did not randomize the selection of venues and times. The interviewer administered questionnaires at: the Moldovan Consulate in the mornings from Tuesday to Friday (opening days), holding interview sessions for the same number of times on each day of the week; at two local Moldovan Orthodox Churches on Sundays, holding interview sessions for the same number of times at each church; at the parking lot usually attended by Moldovans because it is from here that vehicles providing a transport service depart for Moldova on Saturdays; and at the park where Moldovan women tend to meet on Saturdays when the weather is fine. Verbal informed consent was obtained from all participants before their enrolment.

The trained researcher started interviewing 205 women, and the interview was completed with 173 of them. In this article, we analyze a sample of 170 Moldovan women living legally in Italy, having excluded 3 women without documents. At each interviewing session (37 in all), an average of four women refused to participate. Their refusal were mainly due to shortage of time - because they were going to an appointment with the consular officer, for instance, or because they would be late for Mass.

### Questionnaire

The tool used for this study was an ad hoc questionnaire administered to our sample population. It consisted of 55 multiple-choice or open-answer questions administered by a trained interviewer, covering the following domains: socio-demographic factors; lifestyles; perceived health symptoms; self-reported diagnoses of certain specific diseases; and recourse to public health services. The questionnaires were anonymized for the purposes of our analysis (available at https://www.slang-unipd.it/progetti-di-ricerca/migration-and-occupational-health-understanding-the-risks-for-eastern-european-migrant-women/).

The variables measured for each domain were as follows:

1. Socio-demographic factors:

- Age, expressed in years, and grouped as: “20–34”, “35–44”, “45–54”, “55–64” and “> 65” years;
- Schooling, expressed in years of school attended;
- Years living in Italy;
- Employment, classified by risks of exposure for the purposes of this study in 5 groups of occupations as follows: home care-workers (live-in and live-out paid caregivers); cleaners (live-in and live-out domestic workers or industrial cleaners); health care workers (nurses and social care operators); or other occupations (all forms of employment other than those mentioned above); and unemployed;
- Health literacy, measured with the “Single Item Literacy Screener” (SILS), a single-item questionnaire used to identify any impairment in adults’ understanding of health material: “How often do you need to have someone help you when you read instructions, pamphlets or other written material from your doctor or pharmacist?” with 5 possible answers (1. Never; 2. Rarely; 3. Sometimes; 4. Often, 5. Always). Scores higher than 2 indicate some difficulty in understanding the meaning of printed health-related material [16]. Respondents’ scores were used to divide our sample into groups with a high health literacy (SILS questionnaire scores of 1 or 2) or low health literacy (for scores of 3, 4 or 5). A previous study judged that the Italian version of the SILS – as an indicator of limited reading and understanding ability regarding health information – is a better tool for measuring HL than more complex functional HL measurement instruments [17].

2. Lifestyle factors:

- Smoking habit, regardless of the number of cigarettes smoked a day. Ex-smokers were classified as non-smokers for the purposes of this study;
- Drinking habit, expressed as the mean daily alcohol intake measured in units of alcohol (UA), where 1 UA = 12 g ethanol, i.e. approximately 250 ml beer, 75 ml wine or 25 ml spirits;
- Sport or physical exercise in free time in the previous 12 months: rated as “Never”, “Less than once a week”, or “At least once a week”;
- Weight (kg) and height (cm), from which we calculated the respondent’s Body Mass Index (BMI, kg/m^2^), then divided our sample into BMI categories: “Underweight” (BMI less than 18.5); “Normal weight” (BMI between 18.5 and 24.9); “Overweight” (BMI between 25 and 29.9); and “Obese” (BMI over 30).

3. Items concerning self-reported diagnoses were drawn from the Italian version of the “European Health Interview Survey” (EHIS, Eurostat) adopted by the Italian National Institute of Statistics (ISTAT) [18, 19]. The assessment concerned whether any of the following diseases had been diagnosed by the respondent’s doctor: asthma, allergies, bronchitis, myocardial infarction, coronary diseases, hypertension, diabetes, lumbar and cervical disorders, arthritis/arthrosis, depression and anxiety.
4. Items regarding perceived symptoms were extracted from the “Health and Work Performance Questionnaire” (HWPQ) [17]. Specific symptoms were chosen because they are often associated with chronic stress [20] which is reportedly a risk to migrant care workers’ mental health [21]. The questions concerned whether respondents had suffered any of the following symptoms in the previous 6 months: headache, trouble sleeping, fatigue, lack of appetite, difficulty concentrating, gastro-intestinal disturbances, dizziness, shortness of breath, and difficulty relaxing. Respondents answered on a 6-point scale ranging from “Never” (0) to “Daily”, as reported elsewhere [6] [22]. Then they were pooled into three groups (“Never”, “Sometimes” and “Daily”) for our analysis.
5. To assess aspects of health care, we first inquired whether respondents were covered by free medical insurance and social security benefits, and whether they had their own, trusted doctor in Italy or in Moldova. To examine their access to health care services, our questionnaire included other elements drawn from the EHIS [23]. The questions concerned whether respondents had: “seen a GP in the previous four weeks”; “been examined by a specialist in the previous four weeks”; “gone to an Emergency Department in the previous 12 months”; and/or “been hospitalized in the previous 12 months”. Women in the appropriate age groups were also asked if they had undergone mammography and PAP smear/HPV testing at least once in their life (Italian NHS prevention programs recommend mammography screening for women aged 50 to 74, and HPV screening [HPV test or PAP smear] for women aged 25–64).
  Regarding the completeness of the data, there were no missing values for the occupational category variable, but for the items regarding the health literacy measure and age group there were 3 (1.8%) and 6 (3.5%) missing values, respectively. We did not apply any imputation techniques, given the low proportions of cases with missing data.

### Data for comparison

The results of the “European Health Interview Survey 2015” (EHIS) [18] questionnaire administered to a sample of Italian women (*n* = 1827) living in the country’s north-eastern region, and aged between 20 and 74, were compared with the results of the present study on Moldavan migrants.

### Statistical analysis

A descriptive analysis was conducted. We calculated the means and standard deviations for quantitative variables, and the relative and absolute frequencies for categorical variables. Although a convenience time-space method for sampling hard-to-reach populations was applied, we used statistical inference, assuming that this sampling method ensures that the sample is to some degree representative of the entire population. In particular, a chi-squared test was used to test the difference in the distribution of a categorical variable, but Fisher’s exact test was applied when the expected frequencies were < 5. Differences in quantitative measures by group were tested with ANOVA. A backward stepwise logistic regression model with an exit probability of 0.10 was used to test whether access to primary care (the dependent variable was the item “Had you seen a GP in the previous four weeks? Yes/No”) was associated with health literacy, adjusting for sociodemographic variables, health behavior variables and previously-diagnosed diseases. The “R: A language and environment for statistical computing” (R Foundation for Statistical Computing, Vienna, Austria) was used for the analysis [24].

## Results

Table 1 shows the sample’s characteristics. They ranged in age from 21 to 69 years (mean 46.5; SD 12.3). They had attended school for a mean 12.7 years (SD 3.7). The sample’s distribution in the different job categories revealed that 28.2% were home care workers, 27.1% were cleaners, 5.9% were health care workers, 28.8% had other occupations, and 10% were unemployed.

**Table 1:** Characteristics of the sample

Variable		Results (***N*** = 170)
**Socio-demographic variables**		
**Years in Italy**	mean (SD)	12.4 ± 6.2
**Age groups**	n (%)	
20–34		32 (19.5%)
35–44		41 (25.0%)
45–54		36 (22.0%)
55–64		46 (28.0%)
> 65		9 (5.5%)
**Years of education**	mean (SD)	12.7 (3.7)
**Low health literacy: SILS score > 2**	n (%)	21 (13.0%)
**Occupation**	n (%)	
Home care workers		48 (28.2%)
Cleaners		46 (27.1%)
Other		49 (28.8%)
Health care workers		10 (5.9%)
Unemployed		17 (10.0%)
**Health behaviors and anthropometric variables**		
**BMI (kg/m^2)**	mean (SD)	25.6 (4.7)
**Body Mass Index**	n (%)	
Underweight		6 (3.7%)
Normal weight		79 (48.2%)
Overweight		50 (30.5%)
Obese		29 (17.7%)
**Smoking**	n (%)	30 (17.6%)
**Daily alcohol intake (units)**	mean (SD)	1.4 (1.0)
**Sport or physical exercise in the last 12 months**	n (%)	
Never		53 (33.1%)
Less than once a week		21 (13.1%)
Once a week or more		86 (53.8%)
**Health status**		
**Previously diagnosed diseases**		
**Asthma**	n (%)	8 (4.9%)
**Bronchitis**	n (%)	6 (3.7%)
**Myocardial infarction**	n (%)	7 (4.3%)
**Coronary disease**	n (%)	9 (5.5%)
**Hypertension**	n (%)	44 (27.0%)
**Arthritis/arthrosis**	n (%)	56 (34.1%)
**Lumbar disorders**	n (%)	71 (44.4%)
**Cervical disorders**	n (%)	62 (38.3%)
**Diabetes**	n (%)	9 (5.5%)
**Allergies**	n (%)	59 (36.0%)
**Depression**	n (%)	22 (13.7%)
**Anxiety**	n (%)	14 (8.6%)
**Symptoms in the last 6 months**		
**Headache**	n (%)	
Never		32 (19.5%)
Sometimes		58 (35.4%)
Daily		74 (45.1%)
**Trouble sleeping**	n (%)	
Never		71 (43.8%)
Sometimes		27 (16.7%)
Daily		64 (39.5%)
**Extreme fatigue**	n (%)	
Never		78 (47.6%)
Sometimes		23 (14.0%)
Daily		63 (38.4%)
**Lack of appetite**	n (%)	
Never		129 (78.7%)
Sometimes		10 (6.1%)
Daily		25 (15.2%)
**Difficulty concentrating**	n (%)	
Never		92 (56.8%)
Sometimes		37 (22.8%)
Daily		33 (20.4%)
**Gastro-intestinal problems**	n (%)	
Never		91 (55.5%)
Sometimes		27 (16.5%)
Daily		46 (28.0%)
**Dizziness**	n (%)	
Never		76 (46.3%)
Sometimes		51 (31.1%)
Daily		37 (22.6%)
**Shortness of breath**	n (%)	
Never		132 (80.5%)
Sometimes		15 (9.1%)
Daily		17 (10.4%)
**Difficulty relaxing**	n (%)	
Never		95 (58.3%)
Sometimes		23 (14.1%)
Daily		45 (27.6%)
**Use of public health services**		
**Mammography at least once in life**^a^	n (%)	
No		7 (9.6%)
Yes		66 (90.4%)
**Pap smear at least once in life**^b^	n (%)	
No		17 (11.3%)
Yes		133 (88.7%)
**GP visit in previous 4 weeks**	n (%)	78 (47.6%)
**Specialist visit in previous 4 weeks**	n (%)	61 (37.2%)
**Emergency dept. visit in previous 12 months**	n (%)	40 (24.4%)
**Hospitalization in previous 12 months**	n (%)	20 (12.2%)

^a^Only for women of screening age (50–74 years)

^b^Only for women of screening age (25–64 years)

Table 2 shows the bivariate analysis conducted on the variables considered in the questionnaire by age group. There was a high prevalence of allergies and lumbar disorders at all ages, and a high prevalence of depression clustered in the intermediate age groups. Older age was associated with a higher BMI, and with an increase in the reported prevalence of some diseases, such as hypertension, arthritis/arthrosis, cervical disorders, and diabetes.

**Table 2:** Results of bivariate analysis: distribution of different questionnaire variables by age group

		AGE					
		20–34 (***N*** = 32)	35–44 (***N*** = 41)	45–54 (***N*** = 36)	55–64 (***N*** = 46)	> 65 (***N*** = 9)	***p***
**Socio-demographic variables**							
**Year in Italy**		9.7 (4.1)	12.2 (5.8)	13.0 (4.7)	14.6 (7.5)	16.1 (4.3)	0.002
**Occupation**	n (%)						< 0.001
Home care workers		2 (6.2%)	3 (7.3%)	8 (22.2%)	24 (52.2%)	6 (66.7%)	
Cleaners		6 (18.8%)	16 (39.0%)	12 (33.3%)	10 (21.7%)	2 (22.2%)	
Other		20 (62.5%)	17 (41.5%)	2 (5.6%)	9 (19.6%)	0 (0.0%)	
Health care workers		2 (6.2%)	2 (4.9%)	5 (13.9%)	1 (2.2%)	0 (0.0%)	
Unemployed		2 (6.2%)	3 (7.3%)	9 (25.0%)	2 (4.3%)	1 (11.1%)	
**Formal education (years)**	mean (SD)	13.2 (3.0)	13.3 (3.6)	13.2 (2.8)	12.2 (3.1)	14.4 (4.7)	0.273
**Health literacy: SILS score > 2**	n (%)	3 (9.4%)	7 (17.1%)	3 (9.1%)	4 (9.1%)	3 (33.3%)	0.277
**Health behavior variables**							
**BMI (kg/m^2)**	mean (SD)	22.2 (3.2)	24.6 (3.8)	27.0 (5.5)	27.5 (4.1)	27.6 (4.2)	< 0.001
**Body Mass Index**	n (%)						< 0.001
Underweight		2 (6.5%)	2 (4.9%)	0 (0.0%)	1 (2.3%)	0 (0.0%)	
Normal weight		25 (80.6%)	22 (53.7%)	17 (47.2%)	12 (27.9%)	2 (22.2%)	
Overweight		3 (9.7%)	13 (31.7%)	13 (36.1%)	16 (37.2%)	4 (44.4%)	
Obese		1 (3.2%)	4 (9.8%)	6 (16.7%)	14 (32.6%)	3 (33.3%)	
**Smoking**	n (%)	8 (25.0%)	11 (26.8%)	3 (8.3%)	4 (8.7%)	1 (11.1%)	0.063
**Daily alcohol intake (units)**	mean (SD)	1.8 (1.6)	1.3 (0.7)	1.1 (0.4)	1.4 (0.7)	1.1 (0.6)	0.151
**Sport or physical exercise in previous 12 months**	n (%)						0.193
Never		8 (25.0%)	15 (36.6%)	17 (51.5%)	12 (26.7%)	1 (11.1%)	
Less than once a week		3 (9.4%)	6 (14.6%)	2 (6.1%)	8 (17.8%)	2 (22.2%)	
Once a week or more		21 (65.6%)	20 (48.8%)	14 (42.4%)	25 (55.6%)	6 (66.7%)	
**Health status**							
**Previously diagnosed diseases**	n (%)						
**Asthma**		0 (0.0%)	2 (5.0%)	0 (0.0%)	5 (10.9%)	1 (11.1%)	0.075
**Bronchitis**		0 (0.0%)	2 (5.0%)	2 (5.9%)	1 (2.2%)	1 (11.1%)	0.357
**Myocardial infarction**		1 (3.1%)	1 (2.5%)	1 (2.9%)	4 (8.7%)	0 (0.0%)	0.733
**Coronary disease**		1 (3.1%)	0 (0.0%)	4 (11.8%)	4 (8.9%)	0 (0.0%)	0.162
**Hypertension**		3 (9.4%)	6 (15.0%)	8 (23.5%)	19 (42.2%)	5 (55.6%)	0.001
**Arthritis/arthrosis**		4 (12.5%)	6 (15.0%)	11 (32.4%)	28 (60.9%)	7 (77.8%)	< 0.001
**Lumbar disorders**		10 (31.2%)	14 (35.9%)	18 (52.9%)	24 (54.5%)	4 (50.0%)	0.165
**Cervical disorders**		6 (18.8%)	13 (32.5%)	15 (44.1%)	24 (53.3%)	4 (50.0%)	0.025
**Diabetes**		0 (0.0%)	0 (0.0%)	2 (5.9%)	6 (13.3%)	1 (11.1%)	0.024
**Allergies**		10 (31.2%)	17 (42.5%)	14 (41.2%)	14 (30.4%)	4 (44.4%)	0.678
**Depression**		2 (6.5%)	6 (15.0%)	8 (24.2%)	5 (11.1%)	1 (11.1%)	0.318
**Anxiety**		1 (3.1%)	2 (5.0%)	6 (17.6%)	4 (8.9%)	1 (11.1%)	0.249
**Symptoms in previous 6 months**	n (%)						
**Headache**							0.457
Never		6 (18.8%)	6 (14.6%)	4 (11.4%)	12 (26.7%)	4 (44.4%)	
Sometimes		13 (40.6%)	14 (34.1%)	13 (37.1%)	15 (33.3%)	3 (33.3%)	
Daily		13 (40.6%)	21 (51.2%)	18 (51.4%)	18 (40.0%)	2 (22.2%)	
**Trouble sleeping**							0.168
Never		16 (51.6%)	21 (51.2%)	14 (40.0%)	15 (34.1%)	3 (33.3%)	
Sometimes		6 (19.4%)	7 (17.1%)	5 (14.3%)	5 (11.4%)	4 (44.4%)	
Daily		9 (29.0%)	13 (31.7%)	16 (45.7%)	24 (54.5%)	2 (22.2%)	
**Extreme fatigue**							0.077
Never		20 (62.5%)	20 (48.8%)	16 (45.7%)	17 (37.8%)	3 (33.3%)	
Sometimes		6 (18.8%)	8 (19.5%)	3 (8.6%)	4 (8.9%)	2 (22.2%)	
Daily		6 (18.8%)	13 (31.7%)	16 (45.7%)	24 (53.3%)	4 (44.4%)	
**Lack of appetite**							0.651
Never		27 (84.4%)	31 (75.6%)	27 (77.1%)	36 (80.0%)	6 (66.7%)	
Sometimes		1 (3.1%)	4 (9.8%)	1 (2.9%)	4 (8.9%)	0 (0.0%)	
Daily		4 (12.5%)	6 (14.6%)	7 (20.0%)	5 (11.1%)	3 (33.3%)	
**Difficulty concentrating**							0.587
Never		15 (46.9%)	21 (52.5%)	19 (54.3%)	29 (65.9%)	7 (77.8%)	
Sometimes		9 (28.1%)	10 (25.0%)	9 (25.7%)	6 (13.6%)	2 (22.2%)	
Daily		8 (25.0%)	9 (22.5%)	7 (20.0%)	9 (20.5%)	0 (0.0%)	
**Gastro-intestinal problems**							0.245
Never		18 (56.2%)	26 (63.4%)	18 (51.4%)	23 (51.1%)	5 (55.6%)	
Sometimes		9 (28.1%)	5 (12.2%)	7 (20.0%)	4 (8.9%)	2 (22.2%)	
Daily		5 (15.6%)	10 (24.4%)	10 (28.6%)	18 (40.0%)	2 (22.2%)	
**Dizziness**							0.653
Never		15 (46.9%)	20 (48.8%)	15 (42.9%)	20 (44.4%)	5 (55.6%)	
Sometimes		14 (43.8%)	10 (24.4%)	11 (31.4%)	14 (31.1%)	2 (22.2%)	
Daily		3 (9.4%)	11 (26.8%)	9 (25.7%)	11 (24.4%)	2 (22.2%)	
**Shortness of breath**							0.550
Never		29 (90.6%)	33 (80.5%)	29 (82.9%)	32 (71.1%)	7 (77.8%)	
Sometimes		2 (6.2%)	3 (7.3%)	4 (11.4%)	5 (11.1%)	1 (11.1%)	
Daily		1 (3.1%)	5 (12.2%)	2 (5.7%)	8 (17.8%)	1 (11.1%)	
**Difficulty relaxing**							0.077
Never		18 (56.2%)	27 (65.9%)	24 (68.6%)	21 (47.7%)	4 (44.4%)	
Sometimes		9 (28.1%)	2 (4.9%)	3 (8.6%)	7 (15.9%)	2 (22.2%)	
Daily		5 (15.6%)	12 (29.3%)	8 (22.9%)	16 (36.4%)	3 (33.3%)	
**Use of public health services**							
**Mammography at least once in life**^a^							
No		–	–	2 (11.1%)	5 (8.9%)	0 (0.0%)	0.325
Yes		–	–	16 (88.9%)	41 (91.1%)	9 (100.0%)	
**Pap smear at least once in life**^b^							
No		2 (6.9%)	5 (12.2%)	6 (17.6%)	4 (8.7%)	–	0.458
Yes		27 (93.1%)	36 (87.8%)	28 (82.4%)	42 (91.3%)	–	
**GP visits in previous 4 weeks**	n (%)	11 (34.4%)	15 (36.6%)	17 (50.0%)	29 (63.0%)	5 (55.6%)	0.062
**Specialist visits in previous 4 weeks**	n (%)	11 (34.4%)	11 (26.8%)	12 (35.3%)	23 (50.0%)	2 (22.2%)	0.199
**Emergency dept. visits in previous 12 months**	n (%)	8 (25.0%)	8 (19.5%)	6 (17.6%)	14 (30.4%)	3 (33.3%)	0.599
**Hospitalizations in previous 12 months**	n (%)	4 (12.5%)	3 (7.3%)	3 (8.8%)	8 (17.4%)	1 (11.1%)	0.633

^a^*Only for women of screening age (50–74 years)*

^b^*Only for women of screening age (25–64 years)*

**Fig. 1:**
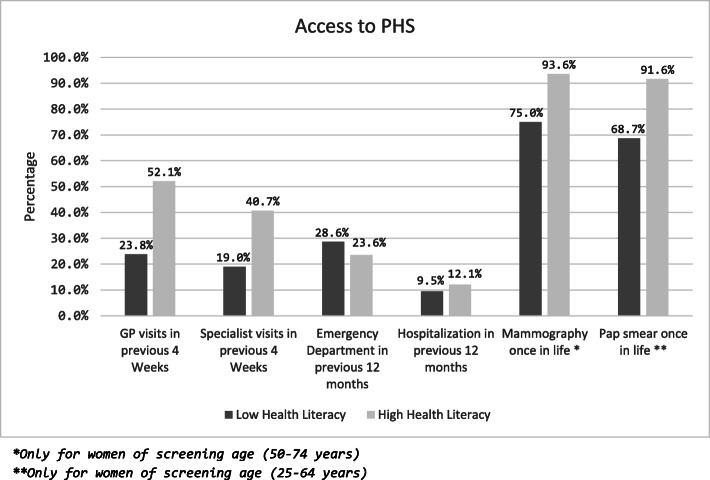
Recourse to public health care services by health literacy level. *Only for women of screening age (50–74 years). **Only for women of screening age (25–64 years)

Table 3 shows the distribution of respondents’ lifestyles and usage of health care services by their health literacy level. The prevalence of active smokers in the low health literacy group was more than twice as high as in the high health literacy group. There was also a significant association between high and low levels of health literacy and respondents’ occupations (p 0.016). In particular, nearly half of respondents with low levels of health literacy were employed as cleaners. There was also evidence of the group with a high health literacy making more visits to GPs (see Fig. 1). The stepwise logistic regression showed that the odd of access to primary care (in the previous 4 weeks) was increased with higher BMI (OR 1.1, 95%C.I. 1.0–1.2) and lumbar disease (OR 2.3 95%C.I. 1.1–4.7), instead was reduced, approaching statistical significance, in case of low levels of health literacy (OR 0.4 95%C.I. 0.2–1.07) (data not shown).

**Table 3:** Results of bivariate analysis: distribution of different questionnaire variables by level of health literacy

		***Low health literacy (N = 21)***	***High health literacy (N = 140)***	***p***
***Socio-demographic variables***				
***Year in Italy***	mean (SD)	13.4 (8.5)	12.67 (5.0)	0.542
***Age group***	n (%)			
*20–34*		3 (15.0%)	29 (20.9%)	0.282
*35–44*		7 (35.0%)	34 (24.5%)	
*45–54*		3 (15.0%)	30 (21.6%)	
*55–64*		4 (20.0%)	40 (28.8%)	
*> 65*		3 (15.0%)	6 (4.3%)	
***Occupation***				
*Home care workers*		8 (22.9%)	39 (29.6%)	0.016
*Cleaners*		16 (45.7%)	29 (22.0%)	
*Other*		4 (11.4%)	44 (33.3%)	
*Health care workers*		3 (8.6%)	7 (5.3%)	
*Unemployed*		4 (11.4%)	13 (9.9%)	
***Formal education (years)***	mean (SD)	12.3 (3.8)	13.2 (3.1)	0.375
**Health behavior variables**				
***Body Mass Index***	n (%)			0.856
*Underweight*		0 (0.0%)	5 (3.7%)	
*Normal weight*		12 (57.1%)	65 (47.8%)	
*Overweight*		5 (23.8%)	42 (30.9%)	
*Obese*		4 (19.0%)	24 (17.6%)	
***Smoking***	n (%)	7 (33.3%)	20 (14.3%)	0.053
***Daily alcohol intake (units)***	mean (SD)	1.5 (1.1)	1.4 (1.0)	0.700
***Sport or physical exercise in previous 12 months***	n (%)			0.338
*Never*		8 (40.0%)	45 (32.4%)	
*Less than once a week*		4 (20.0%)	17 (12.2%)	
*Once a week or more*		8 (40.0%)	77 (55.4%)	
**Health status**				
**Previously diagnosed diseases**	n (%)			
**Asthma**		4 (19.0%)	3 (2.2%)	0.006
***Bronchitis***		2 (9.5%)	4 (2.9%)	0.182
***Myocardial infarction***		0 (0.0%)	6 (4.4%)	0.999
***Coronary disease***		2 (9.5%)	6 (4.4%)	0.291
***Hypertension***		9 (42.9%)	31 (22.8%)	0.061
***Arthritis/arthrosis***		8 (38.1%)	46 (33.6%)	0.805
***Lumbar disorders***		10 (50.0%)	58 (43.3%)	0.633
***Cervical disorders***		10 (47.6%)	50 (37.0%)	0.470
***Diabetes***		1 (4.8%)	6 (4.4%)	0.998
***Allergies***		9 (42.9%)	48 (35.0%)	0.770
***Depression***		5 (23.8%)	15 (11.2%)	0.152
***Anxiety***		3 (14.3%)	9 (6.6%)	0.204
***Symptoms in previous 6 months***	n (%)			
***Headache***				0.864
*Never*		5 (23.8%)	24 (17.3%)	
*Sometimes*		8 (38.1%)	50 (36.0%)	
*Daily*		8 (38.1%)	65 (46.8%)	
***Trouble sleeping***				0.022
*Never*		5 (23.8%)	65 (47.1%)	
*Sometimes*		8 (38.1%)	19 (13.8%)	
*Daily*		8 (38.1%)	54 (39.1%)	
***Extreme fatigue***				0.420
*Never*		9 (42.9%)	67 (48.2%)	
*Sometimes*		5 (23.8%)	18 (12.9%)	
*Daily*		7 (33.3%)	54 (38.8%)	
***Lack of appetite***				0.334
*Never*		16 (76.2%)	110 (79.1%)	
*Sometimes*		0 (0.0%)	10 (7.2%)	
*Daily*		5 (23.8%)	19 (13.7%)	
***Difficulty concentrating***				0.401
*Never*		10 (47.6%)	81 (58.7%)	
*Sometimes*		7 (33.3%)	29 (21.0%)	
*Daily*		4 (19.0%)	28 (20.3%)	
***Gastro-intestinal problems***				0.687
*Never*		13 (61.9%)	76 (54.7%)	
*Sometimes*		4 (19.0%)	23 (16.5%)	
*Daily*		4 (19.0%)	40 (28.8%)	
***Dizziness***				0.864
*Never*		11 (52.4%)	62 (44.6%)	
*Sometimes*		6 (28.6%)	44 (31.7%)	
*Daily*		4 (19.0%)	33 (23.7%)	
***Shortness of breath***				0.738
*Never*		16 (76.2%)	113 (81.3%)	
*Sometimes*		2 (9.5%)	13 (9.4%)	
*Daily*		3 (14.3%)	13 (9.4%)	
***Difficulty relaxing***				0.679
*Never*		11 (52.4%)	82 (59.4%)	
*Sometimes*		4 (19.0%)	18 (13.0%)	
*Daily*		6 (28.6%)	38 (27.5%)

Table 4 shows the figures for the prevalence of various diseases in the sample of 20- to 74-year-old Italian women living in the north-east of the country. When compared with the self-reported prevalence of diseases in the sample of Moldavan women (Table 2), there emerged a higher prevalence of all the diseases considered in the Moldovan women. This was true for all age groups, except in the case of diabetes, for which the Moldovan women reported a higher prevalence only among the 45–54, 55–64 and over 65-year-olds.

**Table 4:** Results of bivariate analysis: 95% confidence intervals (CI) for different “European Health Interview Survey 2015” questionnaire variables in north-eastern Italian women aged 20–74, by age group

	20–34 (***N*** = 343)	35–44 (***N*** = 352)	45–54 (***N*** = 399)	55–64 (***N*** = 386)	> 65 (***N*** = 347)	Total (***N*** = 1827)
**Health status**	% (95% CI)	% (95% CI)	% (95% CI)	% (95% CI)	% (95% CI)	n (%)
**BMI**						
Underweight	13.9% (14, 18.1%)	4.6% (2.7, 7.4%)	3.3% (1.8, 5.5%)	5.7% (3.6, 8.6%)	3.5% (1.8, 6.0%)	110 (6.1%)
Normal weight	76v (65.4, 75.4%)	69.6% (64.4, 74.4%)	66.5% (61.65, 71.1%)	56.4% (51.3, 61.4%)	45.5% (40.2, 50.9%)	1116 (61.7%)
Overweight	12.2% (8.9, 16.1%)	19.4% (15.4, 24.0%)	21.2% (17.2, 25.5%)	23.2% (19.1, 27.8%)	34.3% (29.3, 39.6%)	400 (22.1%)
Obese	3.3% (1.6, 5.8%)	6.4% (4.0, 9.5%)	9.1% (6.4, 12.3%)	14.6% (11.2, 18.6%)	16.7% (12.9, 21.1%)	183 (10.1%)
**Smoking**	28.4% (23.7, 33.5%)	18.9% (14.9, 23.4%)	22.5% (18.5, 26.9%)	21.8% (17.7, 26.3%)	15.0% (11.4, 19.2%)	385 (21.3%)
**Health status**						
**Previously diagnosed diseases**						
Asthma	3.2% (1.6, 5.7%)	3.4% (1.8, 5.9%)	4.0% (2.3, 6.5%)	3.1% (1.6, 5.4%)	2.6% (1.2, 4.9%)	60 (3.3%)
Bronchitis	0% (0, 1.1%)	0.6% (0.1, 2.0%)	3.0% (1.6, 5.2%)	3.1% (1.6, 5.4%)	5.5% (3.3, 8.4%)	45 (2.5%)
Myocardial infarction	0% (0, 1.1%)	0% (0, 1.0%)	0% (0, 0.9%)	0.8% (0.2, 2.3%)	2.6% (1.2, 4.9%)	12 (0.7%)
Coronary disease	0% (0, 1.1%)	0% (0, 1.0%)	0.8% (0.25, 2.2%)	1.8% (0.7, 3.7%)	3.2% (1.6, 5.6%)	21 (1.2%)
Hypertension	0% (0, 1.1%)	2.0% (0.8, 4.1%)	10.1% (7.35, 13.4%)	22.8% (18.7, 27.3%)	40.8% (35.6, 46.2%)	275 (15.1%)
Arthritis/arthrosis	0.6% (0.1, 2.1%)	1.1% (0.3, 2.9%)	8.0% (5.6, 11.1%)	20.5% (16.6, 24.8%)	39.4% (34.2, 44.7%)	252 (13.8%)
Lumbar disorders	5.0% (2.9, 7.8%)	8.3% (5.6, 11.7%)	17.0% (13.5, 21.1%)	22.3% (18.3, 26.8%)	27.8% (23.2, 32.9%)	296 (16.3%)
Cervical disorders	4.7% (2.7, 7.5%)	10.5% (7.5, 14.2%)	19.3% (15.6, 23.6%)	18.7% (14.9, 23.0%)	22.3% (18.0, 27.0%)	279 (15.3%)
Diabetes	0.6% (0.1, 2.1%)	0.3% (0, 1.6%)	0% (0, 0.9%)	4.4% (2.6, 7.0%)	9.0% (6.2, 12.5%)	51 (2.8%)
Allergies	15.8% (12.1, 20.1%)	17.0% (13.2, 21.3%)	17.4% (13.85, 21.5%)	13.5% (10.3, 17.3%)	10.2% (7.2, 13.9%)	269 (14.8%)
Depression	0.9% (0.2, 2.5%)	2.6% (1.2, 4.8%)	4.5% (2.7, 7.0%)	4.4% (2.6, 7.0%)	9.0% (6.2, 12.5%)	78 (4.3%)
Anxiety	1.2% (0.3, 3.0%)	2.0% (0.8, 4.1%)	3.3% (1.7, 5.5%)	1.8% (0.7, 3.7%)	4.6% (2.7, 7.4%)	47 (2.6%)
**Use of public health services**						
**Mammography at least once in** life^a^	–	–	88.7% (85.2, 91.7%)	92.5% (89.4, 94.9%)	93.1% (89.9, 95.5%)	1034 (91.3%)
**Pap smear at least once in life**^b^	66.8% (61.5, 71.7%)	93.2% (90.0, 95.6%)	95.2% (92.7, 97.1%)	93.8% (90.9, 96.0%)	66.8% (61.5, 71.7%)	1299 (87.8%)
**GP visits in previous 4 weeks**	25.9% (21.3, 31.0%)	26.3% (21.7, 31.3%)	35.6% (30.8, 40.6%)	39.1% (34.2, 44.3%)	50.6% (45.1, 56.1%)	628 (35.6%)
**Specialist visits in previous 4 weeks**	20.2% (16.0, 24.9%)	21.0% (16.8, 25.7%)	18.1% (14.45, 22.3%)	24.7% (20.4, 29.3%)	27.4 (22.7, 32.4)	393 (22.2%)
**Hospitalizations in previous 12 months**	4.4% (2.5, 7.2%)	6.1% (3.8, 9.1%)	4.8% (2.9, 7.4%)	7.9% (5.4, 11.1)	10.4 (7.4, 14.1)	121 (6.7%)

^**a**^*Only for women in screening age (50–74 years)* ^b^Only for women in screening age (25–64 years)

## Discussion

As expected, this study showed a higher prevalence of several diseases, such as hypertension and diabetes in older age groups. More interestingly, almost all the illnesses considered showed a higher overall prevalence among the Moldovan migrant women than among the Italian controls. The former made more use of health care services than the latter too. An association also emerged between health literacy level and both lifestyle and recourse to health care services.

Our sample of Moldovan migrant women included a sizable proportion who were overweight (30.5%), or obese (17.7%). These figures are higher than the 22.1% for overweight and 10.1% for obesity among Italian women, but only half the percentages for Moldovan women in their home country, where 60.1% are overweight and 31% are obese [25]. Another finding concerns the clustering of higher BMIs in the older age groups, while the younger migrant women had lower BMIs. This may be a sign of an adaptive effect, with the migrants’ lifestyles, such as their dietary habits, approaching those of their adopted country. Another possible interpretation of this phenomenon, however, is that older women put on weight because of the hard living and working conditions they find in their adopted country.

The answers to our questionnaire indicate that less than one in five Moldavan migrant women are smokers – a proportion almost in line with the Italian reference Fig. (21.3%) - and the prevalence of smokers was similar in all age groups. The Moldovan women’s reported alcohol consumption was moderate-to-high (nearly 1.5 UA a day), and one in three of our respondents exceeded the recommended limit for women (1 UA a day). In its “Global Alcohol Report” for the Republic of Moldova, the WHO indicated that alcohol consumption by women over 15 years old averaged 2 units/day [26]. Our results suggest that our Moldovan migrant women drink less than their counterparts at home, and slightly more than Italian women in the same age range, whose average alcohol consumption is 1 UA a day [27].

Concerning physical exercise, half of our sample reported engaging in some form of physical exercise in their free time, but one in three said they never did so. This level of sedentariness is higher than reported by Moldovans in their own country, which is 24.5% for adults generally.

As for the overall health status of our sample, there was a noticeably higher prevalence of several diseases compared with the Italian reference values. We identified a more than twofold self-reported prevalence of hypertension, arthritis/arthrosis, cervical disorders, diabetes, and allergies, and a threefold prevalence of lumbar disorders, depression and anxiety. If we look at the reported prevalence of allergies (36% for the Moldovan group versus 14.8% for the Italian controls), this may reflect the numerous studies in the literature indicating that migration to a highly-industrialized country favors the development of respiratory allergies in migrants [28, 29]. As regards lumbar and cervical disorders, back pain has been found directly related to mental health disorders and stress in fact stress could contribute to the onset or the persistence of chronic pain [30]. Another plausible explanation for these conditions is work-related, given the large proportion of our respondents who were home care workers and cleaners (jobs that involve the manual lifting of sometimes heavy loads). Analyzing our women by age group, the ratio for the prevalence of Moldovan and Italian women with lumbar disorders declines linearly from 6.3 for the younger women to 1.7 for the older age groups. The same trend could be seen for hypertension, for which the ratio went from 10 for the younger women to 1.3 for the older age groups.

Depression was reported by more than 10% of our Moldovan sample, with a slightly higher prevalence in the intermediate age groups. This is three times higher than the prevalence of 4.3% reported by a sample of 1827 Italian women living in the north-east of the country [19]. This difference is more evident among the younger age groups, the prevalence ratio being 7.2 in the youngest age group and dropping gradually to 1.2 for the older women. When we investigated the issue of anxiety, the prevalence of this condition was a remarkable five times higher in the Moldovan women aged 45–54 and 55–64 than in their Italian counterparts, as opposed to a twofold prevalence in the other age groups. Analyzing symptoms usually associated with anxiety, depression and burnout [21] we found quite a high overall prevalence of daily headache, trouble sleeping, and extreme fatigue, possibly as a direct consequence of underlying stress. These symptoms were distributed throughout our Moldovan sample, with no significant differences between the various age groups considered. This could be also explained by high prevalence of chronic pain as described above, in fact chronic pain could be emotionally stressful [30]. Chronic pain in fact is known to change the levels of stress hormones and these can affect your mood, thinking and behavior. Moreover, chronic pain can affect ability to function at home or work making also difficult to participate in social activities and hobbies, which could lead to decreased self-esteem. In addition, chronic pain could provide sleep disturbances, fatigue, trouble concentrating, decreased appetite. These negative changes can dampen overall mood; and this can result in depression and anxiety. In addition, vulnerability to stress has already been described in migratory groups, especially for Eastern European citizens migrating westwards [31]. The stress of migration per se can lead to depression and anxiety [32] or somatization [33] which are frequently underestimated. Such conditions of malaise can also be carried to the migrants’ home countries when they return. In fact, increasing attention is being paid to what has been called the “Italy syndrome”, which is a sort of psycho-social distress suffered by Eastern European migrant women [34]. The scientific literature on this phenomenon is quite limited, while many newspaper investigations discuss it. Cozzi [35] traces the genealogy of the term showing that it was invented by two Ukrainian psychiatrists, Andriy Kiselyov and Anatoliy Faifrych, who identified a specific medical case affecting women returning from Italy: bad mood, sadness, weight loss, loss of appetite, insomnia, tiredness, loss of motherliness, and split identity.

When questioned about their recourse to health care services, our sample of Moldovan women of all ages reported a large number of visits to GPs and specialists. This can be interpreted as a sign of their integration, and proof of the good functioning of the Italian NHS. The proportions of women reportedly seeing a GP or a specialist in the previous month, or being hospitalized in the previous year were 47.6, 37.2 and 12.2%, respectively. These figures are much higher than those of our Italian controls, which were 35.6, 22.2 and 6.7%, respectively.

The proportion of Moldovan migrant women of suitable screening age who reporting having undergone HPV testing or a PAP smear at least once in their life was much the same as for their Italian counterpart (88.8 vs 87.8%). The cumulative proportions of Moldovan and Italian women who had undergone a mammography at least once in their lives was also very similar (90.1% vs 91.3%) [19].

Our Moldovan migrant women’s health literacy was judged to be good, bearing in mind the potential language barriers imposed by migration, as discussed in the literature [36, 37]. This reflects our sample’s generally high level of formal education, consistently with the reportedly well-functioning school systems of Eastern Europe [38]. The majority of our sample were employed as home care workers, a job that promotes the development of health-related skills. On the other hand, our study findings show that the Moldavan migrant women’s health literacy only partly influenced their lifestyles, mainly as regards smoking (which was twice as prevalent among women with a lower health literacy level). An association between a lower health literacy level and a greater nicotine dependence has already been amply documented in the literature [11, 39–41]. Our study found no association between health literacy and BMI, alcohol consumption or physical exercise. Health literacy was a determinant of primary health care service use, however: respondents with a higher health literacy level visited GPs twice as often as those whose health literacy was limited, while recourse to Emergency Departments and hospitalizations was similar for the two sub-groups. There have been reports of a different, sub-optimal use of health care services by less health-literate individuals, especially in the case of migrants [42, 43].

The main strength of our study lies in that it focuses on migrant Moldovan women, a population rarely considered before. The study also has several limitations, however. First of all, although we made every effort to reduce selection bias by carrying out interviews in different places and at different times in order to meet different kinds of people, our sampling method may have introduced a sort of selection bias as it could not represent women with severe diseases. Face-to-face interviews can also be influenced by an interviewer’s probing or appearing to be judgmental, and by participants being concerned about the confidentiality of their answers, which fosters a social desirability bias [44]. In particular, we noted the tendency of our respondents to deny alcohol consumption, while other questions were answered without any apparent reservations.

The impact of our results can be summarized in two points. First, Moldavan migrants in Italy have a good health literacy level overall [45]; and individuals’ health literacy was confirmed as a determinant of their attitude to smoking and to the use of some health care services [37]. Bearing these findings in mind, adequate programs to improve health literacy in the general population - with a view to promoting healthy lifestyles - would be useful but need not target Moldovan migrants specifically [46, 47]. Second, Moldovan migrant women seem to have more health issues than their Italian counterparts, so this migrant population should be a target of prevention programs - focusing on promoting a healthy amount of physical exercise, for instance, and providing appropriate (biopsychosocial) education on how to prevent lumbar pain.

## Conclusions

The prevalence of some diseases was higher in our sample of Moldovan migrant women than in Italian controls of all ages, but especially among the younger women. Health literacy was associated with migrants’ lifestyles and primary health care service usage, as seen previously for the autochthonous population. Tailored prevention programs and interventions should be designed to address the high prevalence of some diseases among Moldovan migrant women.

## Data Availability

The datasets used and analyzed during the current study are available from the corresponding author on reasonable request.

## Abbreviations

NHS: National health system
ISTAT: Italian institute of statistics
EU: European
HWPQ: Health and work performance questionnaire
EHIS: European health interview survey
SILS: Single item literacy screener
UA: Unit alcoholic
BMI: Body mass index
GP: General practitioner
HPV: Herpes papilloma virus
CI: Confidence interval
ANOVA: Analysis of variance
